# Computational identification and experimental characterization of substrate binding determinants of nucleotide pyrophosphatase/phosphodiesterase 7

**DOI:** 10.1186/1471-2091-12-65

**Published:** 2011-12-16

**Authors:** Abby L Parrill, Irene W Wanjala, Truc Chi T Pham, Daniel L Baker

**Affiliations:** 1 Department of Chemistry and the Computational Research on Materials Institute, The University of Memphis, Memphis, TN 38152, USA; 2 Department of Chemistry, The University of Memphis, Memphis, TN 38152, USA

## Abstract

**Background:**

Nucleotide pyrophosphatase/phosphodiesterase 7 (NPP7) is the only member of the mammalian NPP enzyme family that has been confirmed to act as a sphingomyelinase, hydrolyzing sphingomyelin (SM) to form phosphocholine and ceramide. NPP7 additionally hydrolyzes lysophosphatidylcholine (LPC), a substrate preference shared with the NPP2/autotaxin(ATX) and NPP6 mammalian family members. This study utilizes a synergistic combination of molecular modeling validated by experimental site-directed mutagenesis to explore the molecular basis for the unique ability of NPP7 to hydrolyze SM.

**Results:**

The catalytic function of NPP7 against SM, LPC, platelet activating factor (PAF) and para-nitrophenylphosphorylcholine (pNPPC) is impaired in the F275A mutant relative to wild type NPP7, but different impacts are noted for mutations at other sites. These results are consistent with a previously described role of F275 to interact with the choline headgroup, where all substrates share a common functionality. The L107F mutation showed enhanced hydrolysis of LPC, PAF and pNPPC but reduced hydrolysis of SM. Modeling suggests this difference can be explained by the gain of cation-pi interactions with the choline headgroups of all four substrates, opposed by increased steric crowding against the sphingoid tail of SM. Modeling also revealed that the long and flexible hydrophobic tails of substrates exhibit considerable dynamic flexibility in the binding pocket, reducing the entropic penalty that might otherwise be incurred upon substrate binding.

**Conclusions:**

Substrate recognition by NPP7 includes several important contributions, ranging from cation-pi interactions between F275 and the choline headgroup of all substrates, to tail-group binding pockets that accommodate the inherent flexibility of the lipid hydrophobic tails. Two contributions to the unique ability of NPP7 to hydrolyze SM were identified. First, the second hydrophobic tail of SM occupies a second hydrophobic binding pocket. Second, the leucine residue present at position 107 contrasts with a conserved phenylalanine in NPP enzymes that do not utilize SM as a substrate, consistent with the observed reduction in SM hydrolysis by the NPP7-L107F mutant.

## Background

The nucleotide pyrophosphatase/phosphodiesterase (NPP) enzymes are classified as pyrophosphatases or phosphodiesterases depending on the type of substrates they hydrolyze. NPP1-3 are pyrophosphatases that cleave inorganic phosphates from nucleotides and their derivatives [1]. In contrast, NPP2, NPP6 and NPP7 are phosphodiesterases that hydrolyze phosphodiester bonds in lipids and their derivatives [2,3]. NPP4 and NPP5 are yet to be characterized in terms of substrates and types of activity. NPP2 is the only one of the seven mammalian NPP family members that is known to possess both pyrophosphatase and phosphodiesterase activities [4]. NPP2 is also the only mammalian family member to have been characterized by X-ray crystallography [5,6]. As a phosphodiesterase it exhibits a lysophospholipase D (LPLD) activity, in contrast to NPP6 and NPP7 which exhibit lysophospholipase C (LPLC) activity. Besides these specific activities, NPP family members also exhibit distinct substrate preference profiles but no clear structural basis for the preferences has been proposed [1]. NPP family members are of considerable interest due to their role in biological functions ranging from bone mineralization to cancer [4,7,8]. NPP7 specifically has been linked with anti-inflammatory and anti-tumorigenic activities through its influence on the conversion of SM to ceramide [9-11], however homozygous knockout of NPP7 in mice did not lead to spontaneous tumorigenesis although lipid digestion and absorption was altered [12]. Mutagenic deletion of a segment from NPP7 including H353, a residue involved in chelating one of the essential divalent metal cations, has been identified in the rapidly proliferating colon cancer HT-29 cell line [13].

**Figure 1:**
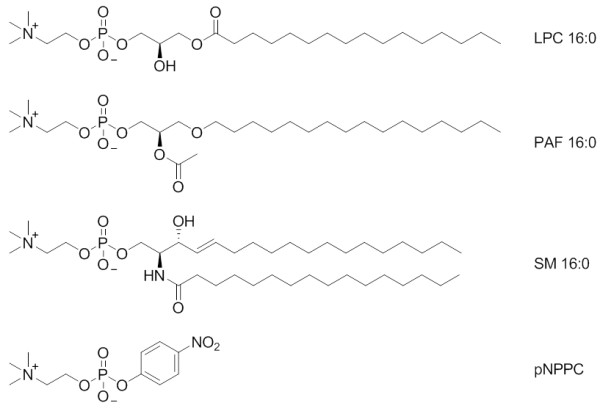
**NPP7 substrate structures**.

NPP7 hydrolyzes lysophosphatidylcholine (LPC, Figure 1), platelet activating factor (PAF, Figure 1) and sphingomyelin (SM, Figure 1) with a lysophospholipase C activity but exhibits a preference for SM over LPC and PAF [10]. Several studies have examined the roles of specific amino acids in the hydrolysis of SM. Three of the conserved metal-chelating residues from the NPP family (D199 and D246 [10,14], as well as H353 [13]) have been mutated to alanine in NPP7, and each mutant was devoid of sphingomyelinase activity. The conserved catalytic threonine residue, T75, has also been mutated to alanine with the expected loss of sphingomyelinase activity [14]. Additional amino acids near the catalytic residue have been mutated in attempts to change substrate specificity to include nucleotide pyrophosphates, including M74K, S76F, and C78N [14]. These mutations all eliminated sphingomyelinase activity without producing pyrophosphatase activity. It is unclear whether the mutated proteins were properly folded, although expression levels were comparable to wild type. More recently, mutations based on a comparative model of NPP7 were performed [15]. In contrast to the result obtained upon mutation of M74 to K, mutation of M74 to L enhanced sphingomyelinase activity, again without gain of pyrophosphatase activity. The model displayed close interaction between F275 and the choline methyl groups, best described as a cation-π interaction. Mutation of F275 to glycine nearly eliminated sphingomyelinase activity. The model displayed no close interaction between F141 and substrates, however, the F141S mutation showed activity nearly as poor as the F275G mutant, indicating either incorrect selection of the conformation of the loop involving residues 140-171, or incorrect substrate positioning. Therefore the interactions between NPP7 and SM are partially defined, and modeled interactions with other substrates have not been supported by experimental results. Further efforts are clearly needed to develop a complete and coherent picture of substrate recognition and discrimination by NPP7.

Lyso-platelet activating factor (lyso PAF), sphingosylphosphorylcholine (SPC), and para-nitrophenyphosphorylcholine (pNPPC, Figure 1) are high affinity substrates for NPP7 (Tables 1, 2, 3 and 4, and [10]). Here we examine the amino acid residues within the NPP7 active site that may be involved in substrate recognition by NPP7. We employed homology modeling to predict protein-substrate interaction points followed by experimental validation of the predictions by mutagenesis and biochemical assays. We used the bacterial *Xac *NPP crystal structure [16] as well as two recently reported NPP2 crystal structures [5,6] to generate homology models of NPP7 for this work. Our modeling results reveal a common choline headgroup binding pocket for all substrates. The strong interactions between the cationic choline and the π electrons of the aromatic sidechain of F275 were easily identified by standard docking algorithms. Experimental mutagenesis results are consistent with the predicted choline headgroup positions for all substrates. The hydrophobic tails proved more challenging to place in the complex. Docking algorithms typically cannot exhaustively search the conformations and positions of long and flexible molecules lacking any polar groups that would be involved in directional interactions such as hydrogen bonding. Manual placement was used, and refinements of hydrophobic tail positions by molecular dynamics were required in order to correlate the substrate complex models with the experimental mutagenesis results in the hydrophobic tail region. The molecular dynamics simulations were important to demonstrate the high mobility of the hydrophobic tail positions, as well as to refine the initial complex of sphingomyelin (SM) to explain the different pattern of experimental mutation impacts on activity for this substrate, relative to the other three examined.

**Table 1:** Kinetic parameters for hydrolysis of LPC 16:0.

Enzyme	K_m _(μM)	V_max _(μM/sec) (× 10^-2^)	k_cat_(sec^-1^)	k_cat_/K_m _(M^-1^sec^-1^) (× 10^4^)
L107F	61 ± 2.8	3.5 ± 0.03	4.2 ± 0.03	6.8 ± 1.6

Y142A	69 ± 0.9	3.0 ± 0.01	3.6 ± 0.01	5.2 ± 0.05

Wild type	57 ± 0.7	2.5 ± 0.01	2.9 ± 0.01	5.1 ± 0.1

E169Q	88 ± 6.8	3.3 ± 0.02	3.8 ± 0.03	4.3 ± 0.4

E169A	100 ± 15	1.0 ± 0.04	1.2 ± 0.05	1.2 ± 0.2

F80A	120 ± 3.0	0.7 ± 0.01	1.4 ± 0.01	1.2 ± 0.3

Y166A	100 ± 5.0	0.9 ± 0.03	1.1 ± 0.03	1.0 ± 0.2

F275A	No activity			

**Table 2:** Kinetic parameters for hydrolysis of PAF 16:0.

Enzyme	K_m _(μM)	V_max _(μM/sec) (× 10^-2^)	k_cat _(sec^-1^)	k_cat_/K_m _(M^-1^sec^-1^) (× 10^4^)
Y142A	75 ± 2	2.0 ± 0.03	2.5 ± 0.04	3.3 ± 0.6

L107F	94 ± 3	2.4 ± 0.3	2.9 ± 0.3	3.1 ± 0.2

Wild type	82 ± 2	1.3 ± 0.02	1.6 ± 0.02	2.0 ± 0.8

E169Q	130 ± 8	1.9 ± 0.1	2.3 ± 0.2	1.7 ± 0.2

F80A	120 ± 3	1.2 ± 0.03	1.4 ± 0.03	1.2 ± 0.4

E169A	95 ± 6	0.8 ± 0.02	0.80 ± 0.02	1.0 ± 0.2

Y166A	200 ± 10	0.7 ± 0.02	0.80 ± 0.04	0.4 ± 0.2

F275A	No activity			

**Table 3:** Kinetic parameters for hydrolysis of SM.

Enzyme	K_m _(μM)	V_max _(μM/sec) (× 10^-2^)	k_cat _(sec^-1^)	k_cat_/K_m _(M^-1^sec^-1^) (× 10^4^)
E169Q	15 ± 5	3.2 ± 0.08	3.8 ± 0.1	25.0 ± 6.0

Y166A	12 ± 0.4	2.6 ± 0.01	3.1 ± 0.2	25.8 ± 5.0

F80A	14 ± 6	2.2 ± 0.3	2.6 ± 0.3	18.6 ± 5.0

Wild type	23 ± 3	3.0 ± 0.1	3.7 ± 0.2	16.5 ± 4.0

E169A	17 ± 2	1.8 ± 0.1	2.2 ± 0.2	11.6 ± 2.0

L107F	37 ± 3	3.5 ± 0.1	4.2 ± 0.1	11.4 ± 4.0

Y142A	47 ± 7	3.1 ± 0.2	3.7 ± 0.2	7.9 ± 3.0

F275A	12 ± 1	0.7 ± 0.1	0.9 ± 0.2	7.5 ± 2.0

**Table 4:** Kinetic parameters for hydrolysis of pNPPC.

Enzyme	K_m _(μM)	V_max _(μM/sec) (× 10^-2^)	k_cat _(sec^-1^)	k_cat_/K_m _(M^-1^sec^-1^) (× 10^4^)
L107F	310 ± 5.0	1.4 ± 0.05	1.7 ± 0.1	0.60 ± 0.02

Wild type	360 ± 8.0	1.1 ± 0.01	1.4 ± 0.1	0.40 ± 0.03

Y142A	400 ± 4.0	1.4 ± 0.01	1.7 ± 0.1	0.40 ± 0.02

F80A	450 ± 10.0	0.63 ± 0.04	0.8 ± 0.1	0.20 ± 0.1

E169A	170 ± 30.0	0.42 ± 0.03	0.5 ± 0.03	0.30 ± 0.02

E169Q	440 ± 5.0	1.2 ± 0.02	1.4 ± 0.1	0.30 ± 0.02

Y166A	820 ± 20.0	0.62 ± 0.03	0.7 ± 0.04	0.09 ± 0.01

F275A	120 ± 5.0	0.23 ± 0.03	0.3 ± 0.03	0.03 ± 0.01

## Methods

### Molecular modeling

Molecular modeling and simulation was done using the Molecular Operating Environment (MOE) software package [17]. Three homology models of NPP7 were built in MOE by aligning the human protein sequence [2] (Genbank code AY20633) with either the bacterial *Xac *crystal structure from the protein data bank (PDB [18] entry 2GSO[16]), the rat NPP2 crystal structure (PDB entry 2XR9[5]) or the mouse NPP2 crystal structure (PDB entry 3NKM[6]). The homology models were energy minimized using the MMFF94x force field, a derivative of the MMFF94 [19] force field with improved treatment of planar nitrogens. Minimizations were terminated when the energy gradient reached a root mean square (RMSG) value of 1 kcal/mol·Å in order to prevent collapse of binding pockets to enhance hydrophobic interactions in the absence of ligand.

Substrate docking with the MOE software was used to study interactions between NPP7 and its previously known substrates and their closely related analogs. Docking used placements of ligand atom triangles into protein site alpha sphere triangles with subsequent focefield refinement using MMFF94x [19]. One hundred initial placements were used in each docking run. pNPPC was directly docked into the active site as a whole molecule since it is small and has limited flexibility. The phospholipid substrates; LPC, PAF, and SM, are highly flexible because of long hydrocarbon chains in their structures. For this reason stepwise docking was done starting with the polar head groups of each substrate. Wall restraints were used to keep the substrate phosphate within 8 Å of the enzyme's active residue, threonine 75. The hydrophobic chains were then extended three carbon atoms at a time followed by energy minimization for the newly added atoms at each step. The protein surface feature within MOE was used to visualize the various channels that could accommodate the substrate hydrophobic tails during binding. Bond rotations were done on the extended chains followed by energy minimization to explore the best positions for each chain and thus the minimum energy conformation for each substrate. The lowest energy conformation was then selected for each substrate.

Molecular dynamics was performed on selected NPP7 substrate complexes using the MOE software. Simulations were performed using the MMFF94x forcefield with the Generalized Born implicit solvation model [20]. The NVT ensemble was used with constraints on bonds to hydrogens. Atomic positions were recalculated at 2 fs timesteps using the Nosé-Poincairé-Andersen equations of motion [21,22]. A heating phase of 100 ps was used to raise the simulation temperature from 0 K to 300 K. The production phase began at the end of the heating phase and continued to the 2000 ps timepoint. Snapshots were saved every 1 ps during both phases of the simulation for analysis.

### NPP7 Plasmid design

A mammalian expression vector pcDNA4/TO/myc His B containing the full-length human NPP7 sequence was a generous gift from Dr. Rui-Dong Duan (Lund University, Sweden). A FLAG-tag and a stop codon were incorporated at position 415 using the sense primer 5'-CCC ATG CTG CAC ACA GAC TAC AAG GAC GAC GAT GAC AAGTAGGAATCTGCTCTTCCG-3' and antisense primer 5'-CGGAAG AGCAGATTCCTACTTGTCATCGTCGTCCTTGTAGTCTGTGTGCAGCATGGG-3'. This generated NPP7ex-FLAG which had previously been demonstrated [23] to retain catalytic activity and localize in the cell culture medium. Mutagenesis was done by PCR using the Quickchange^® ^site directed mutagenesis kit from Qiagen-USA. This customized gene was further sub cloned into the mammalian vector, pcDNA3.1 (+) at the *NotI *and *BamHI *restriction sites. All mutants were generated from this customized plasmid using Qiagen's multi site-directed mutagenesis protocol using only the sense primer. All sense primers used in generating our various mutations are shown in Table 5. All PCR products were amplified in Nova blue E. coli cells and purified according to the manufacturer's protocols (EMD Chemicals).

**Table 5:** Sense primers for hNPP7ex-FLAG mutations.

NPP7 mutation	Sense primer
F80A	TGACCAGCC CTGCCACGCC ACCCTGGTCA CCGGCAAATA

L107F	ACCACCAGCA AGGTGAAG TTC CCCTACCACGCCACGCTGG

Y142A	TGGCTCCTTC TTCGCCCCGG GCGGGAACGT

Y166A	GGCATCGCAC ACAACGCCAAAAATGAGACG

E169A	TCGCAC ACAACTACAAAAATGCGACG GAGTGGAGAG

E169Q	TCGCAC ACAACTACAAAAATGAGACG GAGTGGAGAG

Y194A	ATCTGGTCACACTCGCCTTCGGGGAGCCGGACTCC

F275A	CGGGACATCG AGTTTGAGCT CCTGGACTAC

### NPP7ex-FLAG expression

Human embryonic kidney cells (HEK293T) were seeded in Dulbecco's modified Eagle's medium (DMEM) containing 10% heat inactivated fetal calf serum and 2 mM L-glutamine. The cells were grown overnight at 37°C and 5% CO_2 _up to a 80% confluence before transfection with purified hNPP7ex-FLAG insert subcloned in the pcDNA3.1(+) mammalian vector. Transfection was done in the presence of polyfect transfection reagent from Qiagen according to the manufacturer's protocol. Five to eight hours after trasfection, the culture medium was changed to serum free DMEM and cells incubated for 48 hours at 37°C and 5% CO_2_. Conditioned media containing secreted hNPP7ex-FLAG protein was collected and concentrated using 10,000 molecular weight cutoff (MWCO) filters (Millipore, Billerica, MA, USA) by centrifugation at 3000 × g for 20 minutes. Concentrated NPP7 protein was stored at 4°C in NPP7 assay buffer, pH 8 without addition of adjuvants.

### Western blots

Western blots with the M2 Anti-FLAG antibody (Sigma- Aldrich, St. Louis, MO, USA) were used to verify expression of hNPP7ex-FLAG and its mutants by HEK293T cells. Following Tris-HCl-SDS-PAGE (4-15%) proteins were transferred onto poly-m-vinylidene difluoride (PVDF) membrane (Bio-Rad, Hercules, CA, USA) by electroblotting at 100 V in Tris/Glycine SDS buffer for 1 hour. Nonspecific binding sites were blocked in 50 mM Tris buffered saline (TBST: 2.7 mM KCl, 0.138 M NaCl, 0.5% (v/v) Tween^®^20) and 5% (w/v) non-fat dry milk (NFDM). The membrane was then treated with Anti-FLAG M2 antibody (Sigma-Aldrich, USA) (1:2000 in TBST + 3% NFDM) and anti-goat IgG antibody (Sigma) (1:5000 in TBST + 3% NFDM). Finally, peroxidase-labeled secondary antibodies were used for chemiluminescence detection.

### Normalization and quantification of hNPP7ex-FLAG in CCM

All NPP7 activity assays were performed with concentrated conditioned media (CCM) quantified at least twice by western blot analysis. First, a scanning western blot was run with varying dilutions of the CCM immediately after concentration to assess differences in the amounts of protein in the CCM. Based on the results of the first western blot, all samples were diluted to the same concentration then again verified by a second immunoblot. A known concentration of the carboxy-terminal FLAG-BAP fusion protein from Sigma-Aldrich-USA was included on the second western blot as a standard to quantify the amounts of protein expressed for each sample. All protein bands were normalized relative to the FLAG-BAP standard using the TotalLab software program on a Fotodyne imager (Hartland, WI).

### Assays of NPP7 catalytic activity

All assays of NPP7 catalytic activity were performed three independent times with triplicate samples in each experiment. Standard deviations of results from three independent experiments provide error estimates.

#### Fluorescence-based assay

Hydrolysis of all three lipid substrates by NPP7 was monitored using a modification of Invitrogen's Amplex^® ^Red phosphatidylcholine-specific phospholipase C assay kit. NPP7 assay buffer was prepared as previously described [2] containing 50 mM tris-HCl, pH 8.0, 150 mM NaCl, and 10 mM taurocholic acid but without 2 mM EDTA. Each well on the plate was loaded with a total of 60 μL consisting of 20 μL each of substrate, Amplex red cocktail (10 μM Amplex Red reagent, 0.1 U/mL choline oxidase, 1 U/mL horseradish peroxidase, 4 U/mL alkaline phosphatase) and normalized concentrations of enzyme diluted from CCM. NPP7 activity was monitored by the level of fluorescence using excitation and emission wavelengths of 571 nm and 585 nm, respectively. Data were collected at 1 minute intervals over a 2 hour period in a 96-well, half area plate (Corning Inc., Corning, NY) using a Synergy 2 multi-well plate reader (BioTek, Winooski, VT, USA).

#### Absorbance-based assay

Hydrolysis of pNPPC was monitored using an absorbance assay system based on the *para*-nitrophenolate product that has a maximum absorbance at 405 nm. Each well on the plate was loaded with a total of 60 uL consisting of 20 uL each of NPP7 assay buffer, substrate and normalized amounts of enzyme diluted from CCM. NPP7 activity was monitored by the level of absorbance at 405 nm every minute for a 2 hour period in a 96-well, half area plate (Corning Inc., Corning, NY) using a Synergy 2 multi-well plate reader (BioTek, Winooski, VT, USA).

### Calculation of kinetic parameters

Relative fluorescence and absorbance units were converted into product concentrations. For the fluorescence and absorbance assays, varying concentrations of resorufin or para-nitrophenol were used to generate standard curves. The trend line equation from a plot of standard concentration versus fluorescence/absorbance was used to convert all relative fluorescence and absorbance units to product concentrations. Curve fitting was done with the kaleidograph software program to obtain K_m _and V_max_. K_cat _was calculated using the V_max _obtained and 8.33 nM enzyme concentration used in all assays.

## Results

### hNPP7 homology models

**Figure 2:**
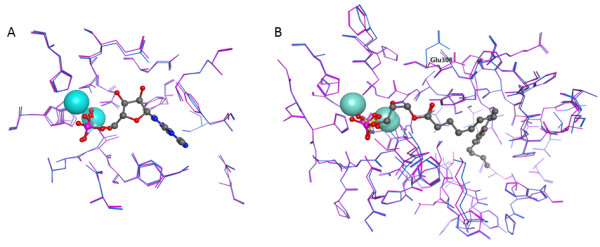
**Comparison of NPP family member crystal structures in the absence (magenta) and presence (blue) of ligand**. A. Comparison of *Xac*. NPP crystal structures 2GSO and 2GSU (with AMP). All-atom RMSD for residues within 4.5 Å of AMP is 0.017 Å. B. Comparison of mouse NPP2 crystal structures 3NKM and 3NKP (with LPA). All-atom RMSD for residues within 4.5 Å of LPA is 0.44 Å. Glu308 is labeled to emphasize the sidechain showing the largest difference between the free and bound forms of the enzyme.

**Figure 3:**
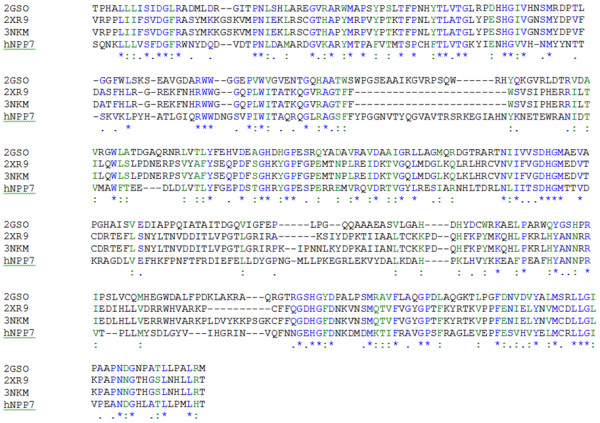
**Alignment of NPP enzyme crystal structures and hNPP7**. Positions with conserved residue identity are shown in blue and marked with asterisks. Positions with conservative substitutions are shown in green and marked with colons.

**Figure 4:**
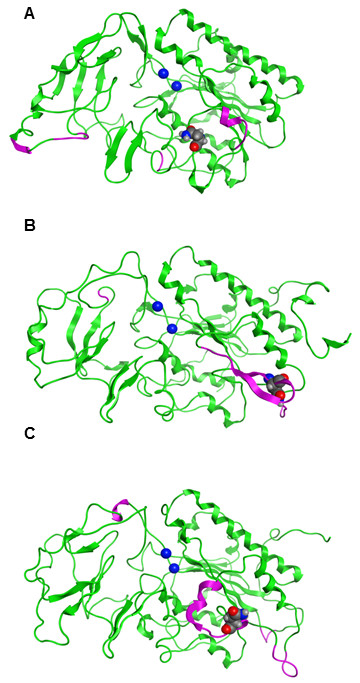
**Comparison of hNPP7 models based on three different template structures**. Green ribbons represent hNPP7 residues aligned against residues in the template. Magenta ribbons represent hNPP7 residues aligned against gaps in the template. Zn^2+ ^ions are shown as blue spheres and the position of N146 is shown as a spacefilling model. A. hNPP7 based on PDB entry 2GSO[16] (*Xac*. NPP), 24.7% amino acid identity. B. hNPP7 based on PDB entry 2XR9[5] (Rat NPP2), 24.9% amino acid identity. C. hNPP7 based on PDB entry 3NKM[6] (Mouse NPP2), 25.3% amino acid identity.

Three homology models of hNPP7 were developed using different crystallized NPP structures. Crystal structures of a bacterial NPP were first reported in 2006 [16]. This year, additional crystallographic structures of NPP2 from two different rodent species became available [5,6]. Notably, the bacterial NPP and mouse NPP2 structures have been crystallized in the presence and absence of products, AMP and LPA, respectively. Figure 2 shows that there is essentially no induced fit of the enzyme to these product structures. The similarity between structures crystallized in the presence and absence of product can be quantitated by heavy-atom root mean square deviation (RMSD) values for atoms within 4.5 Å of the product. The RMSD values are only 0.165 Å for the bacterial NPP and 0.438 Å for the mouse NPP2. Thus either the free or product-bound crystallographic structures should serve equally well as a homology modeling template. Amino acid sequence identity is similar between hNPP7 and the three available NPP structures (24.7-25.3%). However, alignments against the available templates show substantial differences in the number of amino acids that align against gaps in the template structures. The longest stretch of hNPP7 amino acids aligned against gaps in 2GSO [16], 2XR9 [5], and 3NKM [6] were 6, 14, and 14, respectively (Figure 3). Models developed from each template structure are shown in Figure 4, emphasizing regions in the model that aligned against gaps in the template structure as well as the position of N146, a confirmed glycosylation site [14]. The longest gap-aligned stretches all occur in the hNPP7 sequence between P143 and E185. Due to the much smaller gap-aligned segments in the 2GSO-based model of hNPP7, this model was used in all docking studies with substrates. Two prior modeling studies of hNPP7 also used a crystallographic structure of the bacterial NPP as the modeling template [15,24], although one of the studies was published prior to the availability of the NPP2 crystal structures [15]. The insertion of residues in the NPP7 sequence within the segment interacting with LPA in NPP2 limits the utility of the NPP2 crystal structures as templates even though they share a common substrate with NPP7.

### hNPP7 substrate complexes

**Figure 5:**
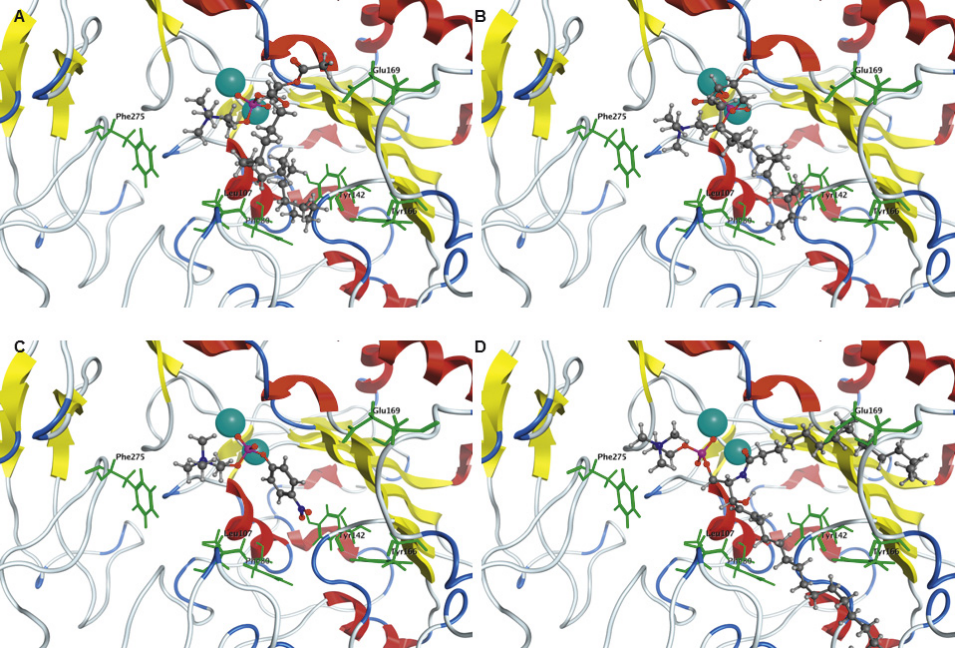
**Comparison of docked substrate positions**. Zn^2+ ^ions are shown as light blue spheres. Substrates are shown as ball & stick models. hNPP7 residues selected for mutagenesis studies are shown as green sticks and labeled. A. PAF 16:0. B. LPC 16:0. C. pNPPC. D. SM.

Three natural NPP7 substrates (PAF 16:0, LPC 16:0, and SM 16:0) and one synthetic substrate (pNPPC) were docked into the model of hNPP7. The natural substrates were flexibly docked with shortened hydrophobic tails and then extended manually. The synthetic substrate was docked flexibly. The docked positions of each substrate are shown in Figure 5 and 5a three dimensional model of all four in complex with the hNPP7 homology model are provided in additional file 1. The four complexes show a common position of the choline headgroup near F275 (cation-π interaction), and a common phosphate group position interacting strongly with one zinc ion (chelated by D199, H203, and H353, shown to the left in all figures) and moderately with the second (chelated by D39, D246 and H247, shown to the right in all figures) (ionic interactions). All four substrates extend from the phosphate group along the bottom edge of the parallel β-sheet bundle (van der Waals interactions). Only the short acetyl sidechain of PAF 16:0 (Figure 1A) and the long palmitoyl chain of SM 16:0 (Figure 1D) extend above the edge of the parallel β-sheet bundle (van der Waals interactions). These complexes were used to select a set of mutation sites to provide experimental feedback and validation of the models. The closest heavy atom distances from each of these selected mutation sites to the docked substrates are shown in Table 6. Based on these complexes, mutation of F275 to a non-aromatic amino acid was expected to result in a less active mutant against all four substrates. L107 was selected for mutation to an aromatic residue with the expectation that this could introduce a favorable cation-π interaction with the choline group, resulting in a more active mutant against all four substrates. However, L107 also makes van der Waals contact with the sphingoid chain of SM 16:0 (3.9 Å) and the more rigid aromatic ring geometry might occlude the space occupied by this chain. Three residues located at various positions near the common position occupied along the bottom edge of the parallel β-sheet bundle were selected for mutation, including F80, Y142, and Y166. One mutation site was selected near the chains extending above the parallel β-sheet bundle, E169. These latter four mutations were expected to show variable differences in activity relative to wild type NPP7 when tested using different substrates due to the variable contact distances displayed in these preliminary docked complexes. For example, Y166 interacted closely with the sphingoid tail of SM 16:0 (3.6 Å) but showed negligible contact with pNPPC (7.4 Å).

**Table 6:** Closest distances (Å) between docked substrates and residues selected for mutation in initial docked complexes.

	PAF 16:0 tail 1-16:0 tail 2-2:0	pNPPC	LPC 16:0	SM 16:0 tail 1-sphingoid tail 2-16:0
**Choline interactions**				

F275	4.5	3.7	3.6	3.8

L107	7.2	7.5	5.6	8.2

**Tail 1 Interactions**				

L107	4.5	6.2	5.1	3.9

F80	6.2	9.5	6.1	7.1

Y166	4.8	7.4	4.8	3.6

Y142	6.9	6.5	6.9	8.8

**Tail 2 Interactions**				

E169	4.0			5.8

### hNPP7 mutations

**Figure 6:**
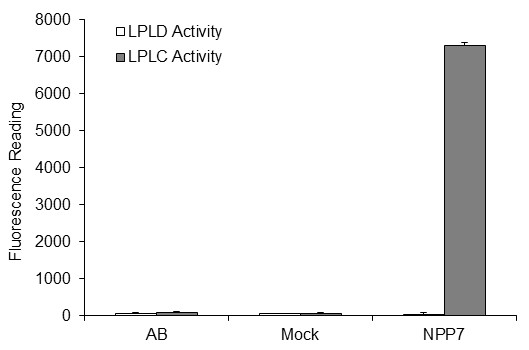
**Fluorescence signals from Amplex Red assays of LPC hydrolysis in the presence (grey bars) and absence (white bars) of alkaline phosphatase using assay buffer (AB) or conditioned media from either mock or NPP7-transfected HEK293T cells**.

**Figure 7:**
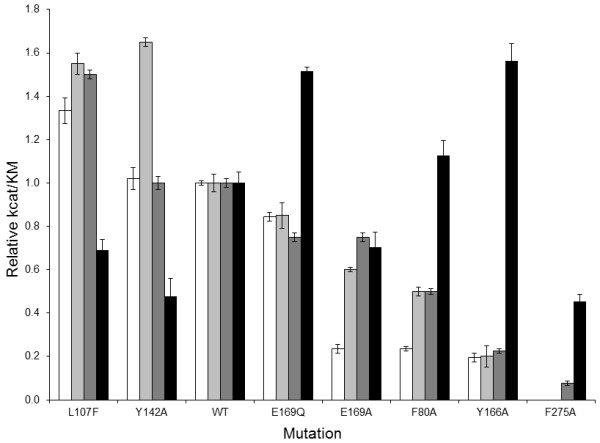
**Relative k_cat_/K_m _values of all mutants for hydrolysis of all substrates**. Substrates include LPC (white bars), PAF16:0 (light grey bars), pNPPC (dark grey bars) and SM (black bars). The relative k_cat_/K_m _values were obtained by dividing the k_cat_/K_m _value of each mutant with that of wild type enzyme against the same substrate.

hNPP7ExFLAG (NPP7-WT) was obtained from the medium of transfected HEK-293T cells without further purification. To ensure that these cells did not produce and export proteins or factors that would interfere with the Amplex Red assay, fluorescence readings using assay buffer (AB), CCM from mock-transfected cells, and CCM from NPP7-transfected cells were compared (Figure 6). Figure 6 demonstrates that only the CCM from NPP7-transfected cells produced a fluorescent signal using the Amplex Red assay, and only when alkaline phosphatase was present to convert phosphocholine produced by LPLC activity to choline. There was no fluorescent signal in the absence of alkaline phosphatase, eliminating any contribution to the fluorescent signal due to direct production of choline by LPLD activity. NPP7-WT and selected mutants were then characterized for their hydrolytic activity against PAF 16:0, LPA 16:0, pNPPC, and SM 16:0. The kinetic parameters obtained from these assays are provided in Tables 1, 2, 3, and 4, and a graphical comparison of relative activity is shown in Figure 7.

Two mutants, F275A and L107F, were selected on the basis of their proximity to the choline headgroup of the substrates (Table 6). Relative to NPP7-WT activities, the F275A mutant showed either no catalytic function (LPA 16:0 and PAF 16:0), a 13-fold reduction in k_cat_/K_m _value (pNPPC) or only a 2.2-fold reduction in k_cat_/K_m _value (SM 16:0). These results are consistent with the models which suggested that the F275A mutation should be detrimental to the recognition and hydrolysis of all substrates. In contrast to the consistently reduced activity of the F275A mutant, the L107F mutant activity differed from WT in a substrate-dependent fashion. The L107F mutant hydrolyzed PAF 16:0, pNPPC, and LPA 16:0 between 1.3 and 1.6 times more effectively than NPP7-WT. Conversely, SM 16:0 was hydrolyzed 1.4 times less effectively by L107F relative to NPP7-WT. These experimental results are consistent with the model-based suggestion that replacement of the nonpolar isobutyl sidechain of leucine with the aromatic sidechain of phenylalanine allowed formation of an additional cation-π interaction with the substrates, but produced crowding against the SM sphingoid tail.

**Figure 8:**
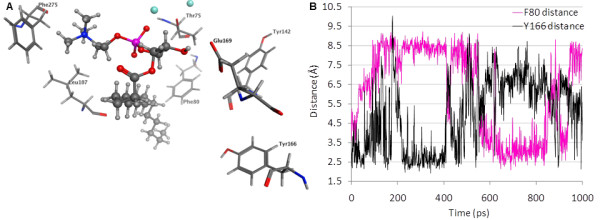
**LPC 16:0 position in NPP7 relative to mutated residues**. Panel A. Structure shown represents an energy-minimized snapshot from the MD simulation. Distances from sidechains to LPC 16:0 are 2.7 Å (E169 to hydroxyl), 2.9 Å (L107 to choline), 3.0 Å (F275 aromatic centroid to choline), 3.0 Å (F80 to nonpolar tail), 7.2 Å (Y166 to nonpolar tail), and 9.0 Å (Y142 to nonpolar tail). The two distances showing the greatest standard deviations when measured at 1 ps intervals during a 2000 ps simulation trajectory were to F80 (2.0 Å) and to Y166 (1.7 Å). Panel B. Molecular dynamics simulations of the LPC 16:0 complex with NPP7 show dynamic motion of the nonpolar tail within the hydrophobic channel as reflected in the distances between the nonpolar tail and the sidechains of F80 and Y166. This dynamic motion results in close interactions at any given time point with either F80 or Y166.

Three mutants, F80A, Y166A, and Y142A, were selected on the basis of their proximity to a hydrophobic tail in all substrates (Table 6). These mutants showed opposing patterns of relative impact on SM relative to the remaining three substrates. The Y142A mutant showed only half the activity of the WT enzyme against SM, but showed similar or better activity against PAF, LPC and pNPPC (Tables 1, 2, 3, and 4). The F80A and Y166A mutants showed reduced activity against LPC, PAF and pNPPC, but showed similar or better activity against SM (Tables 1, 2, 3, and 4). This pattern of activity differences was not predicted by the initial model complexes of these substrates. However, the hydrophobic tails of the endogenous substrates had been placed manually, and exploration of alternate tail positions had been limited. Molecular dynamics simulations of LPC 16:0 and SM were therefore used to allow the substrate tails to explore nearby low-energy placements within the enzyme for a representative substrate from each pattern of activity differences. Molecular dynamics simulations showed a time-dependent alternating interaction of the LPC 16:0 hydrophobic tail with F80 and Y166 based on distances that oscillated between 2.5-3.0 Å and greater than 6.5 Å (Figure 8). This result is consistent with substantially reduced activity due to mutation at either site and suggests that the hydrophobic tail is not restricted to a confined location, which would be an entropically unfavorable binding contribution. Instead, the hydrophobic tail retains considerable flexibility within a broad hydrophobic channel. Molecular dynamics simulations on the SM complex provide an explanation for the opposing impact these mutations had on hydrolysis of SM versus LPC. In particular, the distance of the sphingoid tail to F80 increased during the simulation, stabilizing at a value over 8 Å. This distance is sufficiently high that minimal interactions between SM and F80 occur, consistent with the catalytic activity similar to WT for the F80A mutant when tested using SM as the substrate. The SM sphingoid tail remained within contact distance of Y142 throughout the simulation (averaging 4.5 Å), although the closest contact involved backbone atoms of Y142. The sidechain of Y142 forms contacts between strands of the β-sheet as well as to a neighboring helix. Mutation to a smaller alanine residue may therefore alter the shape or size of the binding pocket for the second tail of SM rather than removing a direct contact. The closest contact of the SM sphingoid tail averaged about 2.5 Å with Y166 with a very low standard deviation of 0.2 Å. A likely explanation for the enhanced activity of the Y166A mutant relative to WT for SM hydrolysis is that greater conformational flexibility of the SM sphingoid tail would be accommodated by the change from a bulky tyrosine to a more compact alanine sidechain, reducing the entropic penalty of transferring SM into the NPP7 binding pocket. Figure 8 shows a snapshot from the simulation emphasizing the close contact with Y166 and distance from F80. The rigid alkene portion of the sphingoid chain sits directly above the bulky sidechain of W119 (not shown to simplify view of mutated residue positions), preventing the sphingoid chain of SM from exercising the same flexibility in the WT as the saturated sidechains of LPC 16:0 and PAF 16:0.

**Figure 9:**
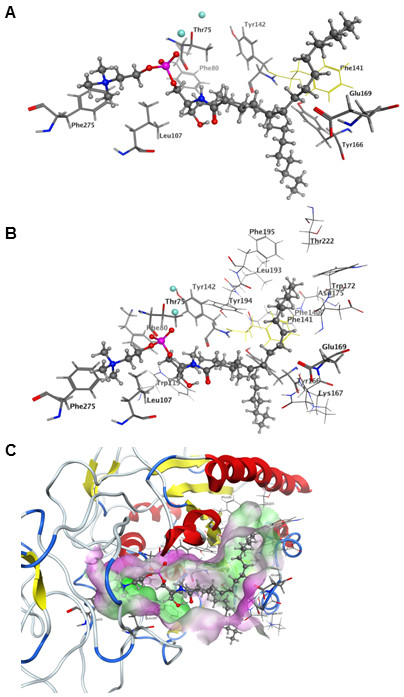
**SM position in NPP7 relative to mutated residues**. Structure shown represents an energy-minimized snapshot from the MD simulation. Distances from sidechains to SM are 2.4 Å (Y166 to sphingoid tail), 2.5 Å (L107 to choline), 2.9Å (E169 to palmitoyl tail), 3.7 Å (F275 aromatic centroid to choline), 5.5 Å (Y142 to sphingoid tail), and 6.9 Å (F80 to sphingoid tail). Mutation of F141 (yellow) to serine was reported by Duan, *et al*. to substantially reduce sphingomyelinase activity of NPP7 [15]. Distance between F141 and the palmitoyl tail is 2.8 Å.

One mutation site, E169, was selected on the basis of isolated contact with the second tail of PAF (short 2:0 chain) and SM (16:0 chain). E169 was mutated to both the similarly-sized glutamine (E169Q) and much smaller alanine (E169A) residue. The mutation to glutamine exhibited minimal differences from WT for LPC, PAF and pNPPC, but showed a 1.5-fold enhanced activity against SM. In contrast, the mutation to alanine showed substantially decreased activity for LPC hydrolysis and less drastic decreases for the remaining substrates. Figure 8 shows that a hydrogen bond between the free glycerol hydroxyl group of LPC 16:0 and E169 forms during the molecular dynamics simulation of the LPC complex. This hydrogen bond could occur in the E169Q but not in the E169A mutant. Thus, the simulation results on the LPC complex with NPP7 are consistent with the observed decrease in activity only of the E169A mutant relative to WT. In contrast to the LPC complex, Figure 9 shows that the interaction of SM with E169 predominantly involves contact between the fatty acyl chain and the E169 sidechain. Thus enhanced activity due to elimination of sidechain ionization in the E169Q mutant and relatively limited activity difference in the E169A mutant are consistent with the simulation results on the SM complex with NPP7.

## Discussion

The choline headgroups of four different substrates are positioned consistently near F275 and L107 in preliminary homology models of NPP7 (Figure 5). The L107F mutant was able to enhance the catalytic function of NPP7 against three of the four substrates tested, with reduced function only against the largest substrate due to steric crowding (Figure 7, and Tables 1, 2, 3, and 4). This finding was consistent with the preliminary models which suggested a cation-π interaction might form in the mutant to enhance interactions with the choline groups of the three smaller substrates (Figure 5). All other human NPP family members have a phenylalanine at the corresponding position. The observed reduction of SM hydrolysis upon mutation of L107 to phenylalanine suggests that the presence of a phenylalanine in the other NPP family members contributes to substrate selectivity by steric interactions with the bulkier SM structure relative to other candidate substrates. This is unlikely to be the sole factor determining whether an NPP enzyme can utilize SM as a substrate as the L107F mutant did not completely lose the ability to hydrolyze SM. The second choline interaction site was probed using an F275A mutant, which showed impaired catalytic function against all four substrates as predicted by the preliminary models (Figure 5 and Tables 1, 2, 3, and 4). This interaction site was previously described by Duan, *et al*, in their modeling study on NPP7. [15] They found that an F275G mutant showed almost no sphingomyelinase activity, consistent with our reduced activity of the F275A mutant. Perhaps surprisingly, an alignment of the mammalian NPP enzymes shows remarkable variability in the residue types that align with F275. Neither NPP2/ATX nor NPP6 have a phenylalanine aligned with F275, despite shared recognition of phospholipids with choline headgroups. NPP2/ATX exhibits D434 at the corresponding position, which may form a favorable ion pair with the cationic choline headgroup instead of the cation-π interaction observed in NPP7. NPP6 aligns Q266 against F275 of NPP7. The role of Q266 in NPP6 will require further investigation, since a strongly favorable interaction with the choline headgroup is unlikely. Notably, both NPP4 and NPP5 have tyrosine residues (260 and 261, respectively) corresponding to F275. Although the endogenous substrates of NPP4 and NPP5 are unknown, the tyrosine sidechain is capable of the same type of cation-π interaction as the phenylalanine sidechain.

**Figure 10:**
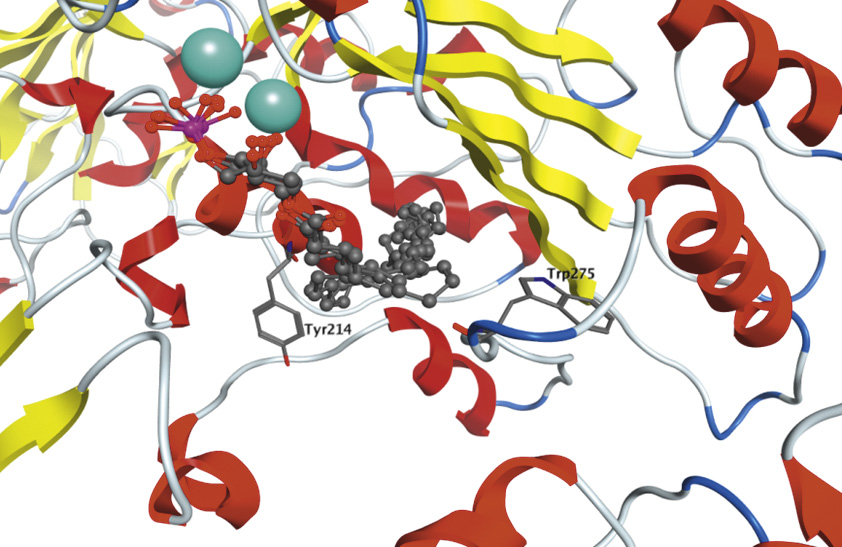
**Crystallographic positions of LPA species in NPP2/ATX (PDB entries **3NKN**, **3NKO**, **3NKP**, **3NKQ**, **3NKR[6]**)**. For clarity, the backbone of only 3NKN is represented as a ribbon colored by secondary structure (yellow for β-sheet, red for α-helix, and blue for turns) and the zinc ions of only 3NKN are represented as cyan-colored spheres. Residues Y214 and W275 are shown as sticks due to the correspondence with F80 and Y166 of NPP7, respectively.

In contrast to the very high consistency between the headgroup position in the model and experimental mutagenesis results, the preliminary modeled positions of the substrate tails were not uniformly consistent with the experimental mutagenesis results. Molecular dynamics studies indicated that the hydrophobic tails showed dynamic interactions within a channel lined with hydrophobic residues. This dynamic behavior explained how mutation of F80 and Y166 to alanine could both have detrimental impact on LPC hydrolysis although they were unable to concurrently interact with the LPC hydrophobic tail (Figures 7 and 8). This finding compares well with the divergent positions of the LPA hydrophobic tails observed in the crystallographic structures of NPP2/ATX (Figure 10) [6]. The hydrophobic tails of LPA species in PDB [18] entries 3NKQ[6] and 3NKR [6] are located near Y214, which corresponds to F80 in NPP7. The hydrophobic tail of the LPA species in PDB entry 3NKP[6] contacts W275, which corresponds most closely in the superposition to NPP7 residue Y166 in the insertion loop first described by Nishimasu, *et al*. in their comparison of the bacterial NPP and NPP2 [6]. The hydrophobic tails of LPA species in PDB entries 3NKN[6] and 3NKO [6] are positioned intermediately between these two sidechains.

The experimental mutation results are compared in Figure 7, and clearly demonstrate a very different pattern for SM relative to LPC, PAF and pNPPC, which show more similar profiles. SM is the only substrate tested that has two long and flexible hydrophobic tails (Figure 1). In our model, these tails occupy completely different binding subpockets, above and below the conserved β-sheet core. This prediction is in direct contrast to the modeling studies of Duan, *et al*., in which both tails were placed into the region below the conserved β-sheet core [15]. In their homology modeling, the loop segment from F140 to E171 was subjected to refinement to generate an ensemble of loop conformations. Conformations were selected for further modeling that kept this lower binding subpocket more open to allow both chains to fit together. Our mutation of E169Q, which showed 1.5-fold enhanced hydrolysis of SM with relatively little impact on hydrolysis of the remaining substrates, suggests that the second tail of SM is not occupying the same site as the hydrophobic tails of the single-tail substrates, as more similarity of mutagenesis results between SM and other substrates would be expected. In fact, we interpret the enhanced activity of the E169Q mutant against SM as a reduction in the charge allowing improved hydrophobic interactions with the second SM tail. Furthermore, Figure 9 shows that the palmitoyl tail of SM makes close van derWaals contact with the sidechain of F141, consistent with poor sphingomyelinase activity observed for an F141S mutant by Duan, *et al*., a finding that was not anticipated based on the common positioning of the hydrophobic tails of SM in their model below the conserved β-sheet core [15]. Based on the current results, the F141S mutant is likely to have activity similar to WT NPP7 against single-tail substrates such as LPC. Importantly, as SM is the optimal substrate for NPP7 out of those tested to date, it is inconsistent to expect that the conformational flexibility of the nonpolar tails would be restricted by common occupation of a pocket of limited size due to the unfavorable entropic contribution that the loss of flexibility would produce. Thus several residues in the longer insertion loop found in NPP7 relative to other mammalian and bacterial NPP family members have been demonstrated to play a role in substrate recognition, and likely contribute to substrate selectivity as proposed by Nishimasu, *et al*. [6] based on their comparison of bacterial NPP and mouse NPP2.

## Conclusions

The current study has defined important differences in recognition of SM and other phospholipid substrates of NPP7. Two contributions to the unique activity of NPP7 against SM have been identified. First is the importance of the amino acid at position 107. NPP7 has leucine at this position although related NPP enzymes have phenylalanine at the corresponding positions. The detrimental impact of the NPP7-L107F mutant on hydrolysis of only SM, compared to the positive impacts on the hydrolysis of LPC, PAF and pNPPC supports our conclusion that this position contributes to substrate selectivity within the NPP family. The substrate-specific impacts of the mutations at position E169 indicate a second contribution to the unique recognition of SM, a second hydrophobic binding pocket for the second hydrophobic tail that is unique to SM.

## List of abbreviations

ATX: Autotaxin; CCM: Concentrated conditioned media; DMEM: Dulbecco's modified eagle's medium; HEK: Human embryonic kidney; LPC: Lysophosphatidylcholine; LPL: Lysophospholipase; MD: Molecular dynamics; MOE: Molecular Operating Environment; MWCO: Molecular weight cutoff; NFDM: Non-fat dry milk; NPP: Nucleotide pyrophosphatase/phosphodiesterase; PAF: Platelet activating factor; PCR: Polymerase chain reaction; PDB: Protein Data Bank; pNPPC: para-nitrophenylphosphorylcholine; PVDM: polyvinylidine difluoride; RMSD: Root mean square deviation; RMSG: Root mean square gradient; SDS-PAGE: Sodium dodecyl sulfate polyacrylamide gel electrophoresis; SM: sphingomyelin; SPC: sphingosylphosphorylcholine; TBST: Tris-buffered saline with TWEEN.

## Authors' contributions

ALP performed the molecular dynamics studies, participated in the development of homology models, contributed to the conception of the study, critically reviewed the analysis of kinetics assays, and completed the manuscript. IWJ participated in the conception of the study, participated in the development of homology models and substrate complexes, performed the kinetic assays, analyzed the kinetic assay results, and wrote an initial partial draft of the manuscript. TCTP participated in the conception of the study, performed the control studies shown in Figure 6, and critically reviewed the analysis of kinetics assay results. DLB participated in the conception of the study and critically reviewed all results. All authors read and approved the final manuscript.

## Supplementary Material

Additional file 1 **Substrate complexes shown in Figure **3. Atomic coordinates of selected homology model and initial docked complexes of four substrates. Click here for file
